# Systematic analysis of global O-serotype prevalence of neonatal extraintestinal pathogenic *Escherichia coli*: A protocol integrating previous research and bioinformatics databases

**DOI:** 10.1371/journal.pone.0354124

**Published:** 2026-08-24

**Authors:** Dongmiao Zhang, Peicen Zou, Ruiqi Xiao, Jiacheng Li, Sihan Sheng, Ruoyi Xiao, Xiao Liang, Yajuan Wang

**Affiliations:** 1 Capital Center for Children’s Health, Capital Medical University, Capital Institute of Pediatrics, Beijing, China; 2 Capital Institute of Pediatrics, Chinese Academy of Medical Sciences & Peking Union Medical College, Beijing, China; Children’s National Hospital, George Washington University, UNITED STATES OF AMERICA

## Abstract

**Background:**

Neonatal extraintestinal pathogenic *Escherichia coli* (ExPEC) severe threat to newborn health. O antigen is a highly variable component of lipopolysaccharide, serves as the main phenotypic marker for serotyping, and represents a promising target for vaccination. The global distribution of O-serotypes in neonatal ExPEC remains unclear, limiting epidemiological understanding and vaccine development.

**Methods:**

This study will integrate literature and genomic data to analyze the global distribution of O-serotypes in neonatal ExPEC systematically. We will systematically search PubMed, Embase, Web of Science, and Scopus for studies reporting neonatal ExPEC serotypes and published between database inception and December 2026. In parallel, neonatal ExPEC genomes will be retrieved from NCBI BioSample, ENA, and CNCB. Strain-level data, including serotype, host age, host disease, isolation site, isolation year, and geography, will be extracted. Serotypes from genomic data will be predicted using multiple bioinformatics tools. The primary outcome is global serotype prevalence; secondary outcomes include temporal trends, geographical variation, and associations with clinical syndromes. Risk of bias will be assessed at both study and strain levels. Descriptive synthesis will integrate literature and genome data, and subgroup or sensitivity analyses will explore heterogeneity if substantial.

**Discussion:**

This study will clarify whether neonatal ExPEC is consistently driven by a limited number of dominant O-serotypes or whether substantial temporal and geographical heterogeneity exists. Defining these patterns is essential for evaluating the feasibility of O-antigen-targeted vaccines, particularly whether broad multivalent strategies or region-specific approaches are more appropriate. By integrating literature and genomic data, this study may also improve understanding of serotype associations with specific invasive syndromes, including sepsis and meningitis. The findings will provide a clearer epidemiological basis for vaccine prioritization, surveillance strategies, and future neonatal ExPEC research in the context of increasing antimicrobial resistance.

**Systematic review registration:**

PROSPERO CRD420251140604

## Introduction

Neonatal infections pose a significant threat to newborn health, [1] with extraintestinal pathogenic *Escherichia coli* (ExPEC) being the dominant pathogen. [2] ExPEC can cause a diverse range of diseases, including sepsis, meningitis, urinary tract infections, and pneumonia. [3] According to 2023 data from the China Antimicrobial Surveillance Network (CHINET), clinically associated *Escherichia coli* (*E. coli*) have a persistently high antimicrobial resistance (AMR) rate, hindering the management of neonatal ExPEC. [4] Hence, there is an urgent need to understand the key virulence factor, lipopolysaccharide (LPS), which is a conserved structure exclusively carried by Gram-negative bacteria. [5]

LPS typically consists of three structural regions: lipid A, core oligosaccharide, and the O antigen. [6] In contrast to the highly conserved lipid A and core oligosaccharide regions, the O antigen exhibits remarkable structural variability. Thus, the O antigen serves as a primary phenotypic typing scheme of *E. coli* in epidemiological traceback. [7] In contrast, conventional laboratory diagnosis of E. coli infections mainly relies on culture-based identification and antimicrobial susceptibility testing. [8] Although recent advances in rapid diagnostic technologies have improved turnaround times, these approaches primarily focus on pathogen identification and resistance profiling rather than characterization of population-level serotype epidemiology. [9] Specific O-serotypes are strongly associated with invasive ExPEC disease in neonates, suggesting that a restricted number of serotypes account for the majority of clinical cases. [10] In addition, O-antigen represents a highly promising vaccine target, making it possible to the development of non-antibiotic therapeutic strategies. [11] Conjugate vaccines targeting prevalent O-serotypes could provide broad protection, similar to the successful model of *pneumococcal* conjugate vaccines. [12] However, the distribution of ExPEC serotypes varies geographically and temporally, and comprehensive knowledge of global serotype epidemiology in neonates is lacking.

Agglutination tests with specific antisera were the traditional gold standard for serotyping. However, it is hampered by its cumbersome, low throughput, subjective interpretation, and the limited availability of antisera for rare serotypes. The emergence of high-throughput next-generation sequencing (NGS) has effectively addressed these limitations. [13] Since the O antigen is typically encoded by conserved housekeeping genes (such as *wzx* and *wzy*), it can be directly determined from whole-genome sequencing (WGS) data, significantly streamlining large-scale serotyping efforts. [14]

Previous studies on neonatal ExPEC serotypes have mostly been single-center or regional investigations, often limited by small sample sizes and heterogeneous methodologies. [15] Some studies have reported ExPEC isolates, but most have no or limited neonatal data. [16] In addition, while many neonatal ExPEC isolates have now been sequenced, serotype data are often absent from publications or databases, limiting their utility for epidemiological synthesis. [17] To date, no systematic review and meta-analysis has comprehensively evaluated the global distribution of ExPEC serotypes in neonates.

This study aims to systematically retrieve both published literature and publicly available genomic data to establish a unified strain-level dataset and to describe the global distribution of O-serotypes among neonatal ExPEC. This protocol will provide a standardized, openly available, and reproducible search and analysis script, including stratified serotype distribution profiles by region, time period, and infection type. By combining published serotyping results with predictions from whole-genome sequencing, we will generate the most extensive global overview to date, informing vaccine development and public health strategies. These resources will provide baseline evidence for neonatal ExPEC surveillance networks, vaccine prioritization, and future multicenter epidemiological investigations.

## Methods and analysis

### Study design and registration

While this protocol incorporates both published literature and publicly available genomic data, it will be conducted in accordance with the Preferred Reporting Items for Systematic Review and Meta-Analysis Protocols (PRISMA-P) guidelines to ensure methodological rigor and transparency. [18] The PRISMA-P 2015 checklist is provided as S1 Checklist. The study protocol has been prospectively registered in the International Prospective Register of Systematic Reviews (PROSPERO) under the unique identifier CRD420251140604. The study is anticipated to be conducted between January 2027 and March 2027. No data extraction, genome retrieval, or statistical analysis has been initiated at the time of submission.

### Ethics approval and consent to participate

Ethics approval is not required for this study, as all data included in this study are derived from previously published studies and publicly available genomic data, and no direct involvement of human participants is anticipated. Findings will be disseminated through national and international academic conferences and published in a peer-reviewed journal.

### Search strategy for literature

We will conduct a comprehensive search of databases, including PubMed, Embase, Web of Science, and Scopus, for studies published between database inception and December 2026. The search strategy will combine terms related to *Escherichia coli*, serotype, genome, and infection type. The search strategy will be adapted for each database. The detailed search strategy for PubMed is provided in S1 File. The PubMed filter for human studies will be applied. We will also manually screen the reference lists of included studies and relevant reviews to identify additional eligible articles.

### Inclusion and exclusion criteria

For this study, neonatal ExPEC will be defined as Escherichia coli isolates recovered from extraintestinal infections in neonates (≤28 days), including bloodstream infections, meningitis, pneumonia, urinary tract infections, and other extraintestinal infections. Studies will be included if they meet the following criteria: (1) reporting E. coli serotypes from neonates (≤28 days) with extraintestinal infections, including sepsis, meningitis, pneumonia, and urinary tract infection; (2) observational studies (cohort, case-control, cross-sectional) and surveillance reports; and (3) data specific to neonates for studies with mixed-age populations.

Studies will be excluded if they meet the following criteria: (1) manuscripts without primary data; (2) studies without serotype data or without access to genomic sequences; and (3) experimental studies not involving clinical isolates.

### Genome acquisition and serotyping

We will retrieve neonatal ExPEC genomes from the National Center for Biotechnology Information (NCBI) BioSample (https://www.ncbi.nlm.nih.gov/biosample), European Nucleotide Archive (ENA) (https://www.ebi.ac.uk/ena/browser/), and China National GeneBank Database (CNCB) (https://www.cncb.ac.cn/). Different search strategies will be adapted for each database. The search strategy for NCBI Biosample is provided in S1 File. The metadata of each result will be fully exported. The samples will be filtered using the “host age” field, with samples that lack such information excluded. The filtering process will be automated with Python code and verified by a researcher. Sequencing results will be downloaded after merging and deduplicating samples identified from both the literature search and the BioSample database search. Serotype prediction will be cross-validated using multiple bioinformatics tools, including SerotypeFinder [19] and ECTyper [20], to ensure accuracy and consensus. Consensus results across tools will be considered as the final serotype assignment; in case of discrepancies, results and original genome will be manually reviewed.

### Data extraction

Two well-trained reviewers will independently extract article data using a standardized form to evaluate eligibility. Any differing opinions will be resolved through discussion and consensus. At the study level, the following information will be collected: title, abstract, first author, publication year, country/region, study design, study period, and sample size.

Subsequently, data will be extracted at the strain level, including O-serotype, host age, clinical syndrome, isolation site, year of isolation, and geographical origin. Where available, additional information will be collected on antimicrobial resistance phenotypes, resistance genes, major virulence determinants, mortality, severe clinical outcomes, and host or clinical risk factors associated with infection. Samples with missing data in specific fields will be systematically identified and excluded only from the analyses requiring those variables, while remaining eligible for other analyses.

### Outcomes

The primary outcome is the pooled global prevalence of *E. coli* serotypes causing neonatal ExPEC infections.

Secondary outcomes will include: (1) temporal trends in serotype distribution; (2) geographical variation in serotype distribution; (3) patterns of association between specific serotypes and clinical syndromes, including infection type, mortality and other severe clinical outcomes; (4) risk factors associated with different serotypes; (5) distribution of antimicrobial resistance phenotypes and/or resistance genes among major O-serotypes; and (6) distribution of major virulence determinants among major O-serotypes, where available.

### Risk of bias assessment

The risk of bias will be assessed at two levels. For studies retrieved from the literature, risk of bias will be evaluated using standardized tools designed for observational studies, such as the Newcastle-Ottawa Scale (NOS). [21] The assessment will consider the selection of participants, the comparability of groups, and the ascertainment of outcomes. Each study will be graded as low, moderate, or high risk of bias.

All included isolates, regardless of whether they originate from published studies or genomic databases, will undergo quality evaluation. Key aspects will include completeness of metadata (host age, clinical syndrome, site of isolation, and geographical origin), clarity of serotype assignment, and sequencing reliability (where applicable). Strains lacking essential metadata or showing ambiguous serotype prediction will be excluded from specific analyses but listed in supplemental materials. This dual approach will ensure both methodological rigor at the study level and reliability at the isolate level, thereby enhancing the robustness of pooled serotype distribution estimates.

If substantial or considerable heterogeneity is identified at the study level or strain level, additional subgroup and sensitivity analyses will be conducted to explore potential influence. [22]

### Data synthesis and analysis

Given the descriptive and mapping nature of this study, no formal meta-analysis will be performed. Instead, we will conduct comprehensive descriptive analyses of the included isolates. The prevalence of O-serotypes will be summarized as proportions with 95% confidence intervals, stratified by geographical region, year of isolation, and clinical syndrome. Distribution patterns will be visualized using frequency tables, bar plots, and geographical maps.

Comparisons across subgroups, including regions, time periods, and infection types, will be performed using chi-square tests or trend analyses where appropriate. For genomic data, serotype predictions will be integrated with literature-based results after deduplication to generate a unified serotype distribution dataset. This integrated dataset will serve as the basis for global mapping and identification of dominant neonatal ExPEC serotypes.

## Discussion

Neonatal ExPEC remains a major cause of invasive infection, yet preventive strategies are constrained by incomplete understanding of which O-serotypes consistently drive disease burden across settings. Although specific serotypes such as O1, O2, O6, and O25 are frequently reported, [11] existing evidence is largely derived from regional or mixed-age studies, making it difficult to determine whether these patterns truly reflect global neonatal epidemiology or local sampling bias. Because serotype prevalence may vary by geography, healthcare infrastructure, and time period, fragmented evidence may limit both epidemiological interpretation and rational vaccine prioritization. Unlike broader ExPEC populations, neonatal infections represent a distinct clinical context shaped by vertical transmission, immature immunity, and severe invasive presentations such as sepsis and meningitis. However, neonatal-specific data are often underpowered, inconsistently reported, or embedded within larger datasets without extractable subgroup information. Integrating literature with publicly available neonatal genomes may help reduce these limitations by expanding strain representation beyond conventional published cohorts.

The major clinical importance of defining neonatal ExPEC serotype distribution lies in vaccine development. O-antigen-targeted vaccines depend on whether a restricted set of serotypes accounts for a substantial proportion of neonatal disease, similar to successful conjugate vaccine strategies used for other bacterial pathogens. If neonatal invasive ExPEC is concentrated within a limited serotype spectrum, this would strengthen the feasibility of targeted multivalent vaccines; conversely, marked regional heterogeneity may indicate the need for geographically tailored strategies. This work may also clarify whether observed serotype differences are linked to infection syndromes such as meningitis versus sepsis, which could suggest distinct pathogenic pathways and improve biological understanding of neonatal ExPEC. Such findings may support future integration of serotype surveillance with virulence, resistance, and mobile genetic element studies.

By systematically consolidating global neonatal data and identifying dominant or variable serotype patterns, this review may provide a clearer epidemiological basis for vaccine antigen selection, surveillance priorities, and future neonatal ExPEC research, particularly in the context of rising antimicrobial resistance where non-antibiotic prevention strategies are increasingly important.

## Supporting information

S1 Checklist PRISMA-P 2015 checklist. (DOCX)

S1 File Search strategy. (DOCX)
