# Supplementary material for: Systematic analysis of global O-serotype prevalence of neonatal extraintestinal pathogenic Escherichia coli: A protocol integrating previous research and bioinformatics databases
Source: PLoS One. 2026 Aug 24;21(8):e0354124. doi: 10.1371/journal.pone.0354124 (PMC13502645; doi:10.1371/journal.pone.0354124)
Supplement: Supplementary file 2 [file pone.0354124.s002.docx]

Search Strategy

#1 "Escherichia coli" OR "E. coli"

#2 "sepsis" OR "bloodstream" OR "meningitis" OR "pneumonia" OR "urinary tract infection" OR "extraintestinal" OR "ExPEC"

#3 "serotype" OR "serotyping" OR "O antigen" OR "O-antigen"

#4 "whole genome" OR "WGS" OR "sequencing" OR "sequence data" OR "genomic epidemiology" OR "assembly" OR "genome database"

#5 #1 AND #2 (For NCBI Biosample)

#6 #3 OR #4

#7 #5 AND#6 (For Pubmed)
