# Supplementary material for: Systematic analysis of global O-serotype prevalence of neonatal extraintestinal pathogenic Escherichia coli: A protocol integrating previous research and bioinformatics databases
Source: PLoS One. 2026 Aug 24;21(8):e0354124. doi: 10.1371/journal.pone.0354124 (PMC13502645; doi:10.1371/journal.pone.0354124)
Supplement: Supplementary file 3 [file pone.0354124.s004.docx]

**Response to Reviewers**

PONE-D-26-22669

Systematic analysis of global O-serotype prevalence of neonatal extraintestinal pathogenic Escherichia coli: a protocol integrating previous research and bioinformatics databases

Thank you for submitting your manuscript to PLOS ONE. After careful consideration, we feel that it has merit but does not fully meet PLOS ONE’s publication criteria as it currently stands. Therefore, we invite you to submit a revised version of the manuscript that addresses the points raised during the review process.

Reply: Thank you for the opportunity to revise our manuscript. We appreciate the constructive comments provided by the Academic Editor and reviewers. We have carefully considered all suggestions and revised the manuscript accordingly. Detailed responses are provided below, and all changes have been highlighted in the tracked-changes version of the manuscript.

**ACADEMIC EDITOR'S COMMENTS:**

In the Introduction, the authors are encouraged to introduce the status quo of identification and AST testing for E. coli, the limitations of conventional diagnostic workflows, as bacterial identification and subsequent AST remain time-consuming processes. However, there are some recent advances in novel diagnostic technologies. Lin et al. recently developed a metabolism-driven colorimetric "Read-to-Answer" sensor array for rapid bacterial identification and AST for different bacteria, including E. coli. More references should be cited, with this one (PMID: 40737437) as an example (citing is optional).

Reply: We thank the Academic Editor for this valuable suggestion. We have revised the Introduction to provide additional background on conventional E. coli identification and antimicrobial susceptibility testing (AST) workflows, highlighting their limitations in terms of turnaround time and laboratory requirements. We have also added a brief description of recent advances in rapid diagnostic technologies and cited the recommended reference (PMID: 40737437), together with other relevant literature. These revisions can be found in the Introduction section of the revised manuscript. (**See in line 60**)

**Journal Requirements:**

When submitting your revision, we need you to address these additional requirements.

1. Please ensure that your manuscript meets PLOS ONE's style requirements, including those for file naming. The PLOS ONE style templates can be found at

https://journals.plos.org/plosone/s/file?id=wjVg/PLOSOne_formatting_sample_main_body.pdf and

https://journals.plos.org/plosone/s/file?id=ba62/PLOSOne_formatting_sample_title_authors_affiliations.pdf

Reply: Thank you. We have carefully reviewed the manuscript and revised the formatting to comply with all PLOS ONE style requirements, including file naming conventions.

2. Thank you for stating the following financial disclosure:

“This study was supported by the National Natural Science Foundation of China (Grant No. 82574169), Beijing Nova Programme Interdisciplinary Cooperation Project (Grant No. 20240484724), and High-level Public Health Technical Personnel Construction Project of Beijing Municipal Health Commission (Grant No. Academic leader -03-02).”

Please state what role the funders took in the study. If the funders had no role, please state: "The funders had no role in study design, data collection and analysis, decision to publish, or preparation of the manuscript."

If this statement is not correct you must amend it as needed.

Please include this amended Role of Funder statement in your cover letter; we will change the online submission form on your behalf.

Reply: We have added the following statement to the cover letter and request that it be updated in the online submission system: This study was supported by the National Natural Science Foundation of China (Grant No. 82574169), Beijing Nova Programme Interdisciplinary Cooperation Project (Grant No. 20240484724), and High-level Public Health Technical Personnel Construction Project of Beijing Municipal Health Commission (Grant No. Academic leader -03-02). The funders had no role in study design, data collection and analysis, decision to publish, or preparation of the manuscript.

3. Please note that funding information should not appear in any section or other areas of your manuscript. We will only publish funding information present in the Funding Statement section of the online submission form. Please remove any funding-related text from the manuscript.

Reply: Thank you. We have reviewed the manuscript and removed all funding-related information from the main text. Funding information is now provided only in the Funding Statement section of the submission system.

4. Your ethics statement should only appear in the Methods section of your manuscript. If your ethics statement is written in any section besides the Methods, please move it to the Methods section and delete it from any other section. Please ensure that your ethics statement is included in your manuscript, as the ethics statement entered into the online submission form will not be published alongside your manuscript.

Reply: Thank you. We have reviewed the manuscript and ensured that the ethics statement appears only in the Methods section.

5. If the reviewer comments include a recommendation to cite specific previously published works, please review and evaluate these publications to determine whether they are relevant and should be cited. There is no requirement to cite these works unless the editor has indicated otherwise.

Reply: Thank you. We reviewed the recommended publication and considered its relevance to our study. Relevant references have been incorporated where appropriate.

6. Please review your reference list to ensure that it is complete and correct. If you have cited papers that have been retracted, please include the rationale for doing so in the manuscript text, or remove these references and replace them with relevant current references. Any changes to the reference list should be mentioned in the rebuttal letter that accompanies your revised manuscript. If you need to cite a retracted article, indicate the article’s retracted status in the References list and also include a citation and full reference for the retraction notice.

Reply: Thank you. We have carefully reviewed and updated the reference list to ensure completeness and accuracy. No retracted articles were cited in the manuscript.

**Reviewers' comments:**

**6. Review Comments to the Author**

Reviewer #1: In the data sets to be analysed, include the comparative analysis of the mortality rates, AMR burden among the serotypes in the study with other serotypes. Include the risk factor analysis for acquisition of such serotypes.

Reply: We thank the reviewer for this valuable suggestion. In response, we have expanded the study outcomes and data extraction framework. Specifically, we have added mortality and other severe clinical outcomes, host and clinical risk factors associated with major O-serotypes, and antimicrobial resistance phenotypes and/or resistance genes as secondary outcomes. Corresponding variables will be extracted where available from published studies and genomic databases. These additions will provide a more comprehensive characterization of neonatal ExPEC epidemiology and strengthen the clinical relevance of the study. (**See in line 142-144, 149-152**)

Reviewer #2: Strengths: Strong rationale, clinically important question, PROSPERO registration, integration of systematic review and genomic epidemiology.

Key weaknesses: Limited statistical framework, insufficient ExPEC definition (The protocol states it will include "neonatal ExPEC" isolates but does not clearly specify which definition will be used ) , inadequate consideration of AMR and virulence factors.

Most important gap to address before publication: Expand the protocol to include genomic characterization beyond O-serotyping (particularly antimicrobial resistance genes and major virulence determinants), as these factors are central to neonatal ExPEC epidemiology and vaccine development.

Reply: We sincerely thank the reviewer for the constructive comments and positive evaluation of our study. Several revisions have been made accordingly.

First, we have clarified the definition of neonatal ExPEC in the Inclusion and Exclusion Criteria section. For this study, neonatal ExPEC is defined as Escherichia coli isolates recovered from extraintestinal infections in neonates (≤28 days), including bloodstream infections, meningitis, pneumonia, and urinary tract infections. (**See in line 105**)

Second, we agree that antimicrobial resistance and virulence characteristics are important components of neonatal ExPEC epidemiology. Therefore, we have expanded the secondary outcomes to include the distribution of antimicrobial resistance phenotypes and/or resistance genes, as well as major virulence determinants among predominant O-serotypes, where such information is available from published studies or genomic databases. These additions complement the primary objective of characterizing global O-serotype distribution while maintaining the overall scope and feasibility of the protocol. (**See in line 142-144, 149-152**)
