# Supplementary material for: Systematic analysis of global O-serotype prevalence of neonatal extraintestinal pathogenic Escherichia coli: A protocol integrating previous research and bioinformatics databases
Source: PLoS One. 2026 Aug 24;21(8):e0354124. doi: 10.1371/journal.pone.0354124 (PMC13502645; doi:10.1371/journal.pone.0354124)
Supplement: Peer review history

# Peer review: Decision Letter 0

**DOI:** 10.1371/journal.pone.0354124.r001

**Authors:** Benjamin Liu

11 Jun 2026

Dear Dr. Zhang,

**ACADEMIC EDITOR'S COMMENTS:**

Please submit your revised manuscript by Jun 26 2026 11:59PM. If you will need more time than this to complete your revisions, please reply to this message or contact the journal office at plosone@plos.org. When you're ready to submit your revision, log on to https://www.editorialmanager.com/pone/ and select the 'Submissions Needing Revision' folder to locate your manuscript file.

- A letter that responds to each point raised by the academic editor and reviewer(s). You should upload this letter as a separate file labeled 'Response to Reviewers'.
- A marked-up copy of your manuscript that highlights changes made to the original version. You should upload this as a separate file labeled 'Revised Manuscript with Track Changes'.
- An unmarked version of your revised paper without tracked changes. You should upload this as a separate file labeled 'Manuscript'.

If you would like to make changes to your financial disclosure, please include your updated statement in your cover letter. Guidelines for resubmitting your figure files are available below the reviewer comments at the end of this letter.

If applicable, we recommend that you deposit your laboratory protocols in protocols.io to enhance the reproducibility of your results. Protocols.io assigns your protocol its own identifier (DOI) so that it can be cited independently in the future. For instructions see: https://journals.plos.org/plosone/s/submission-guidelines#loc-laboratory-protocols. Additionally, PLOS ONE offers an option for publishing peer-reviewed Lab Protocol articles, which describe protocols hosted on protocols.io. Read more information on sharing protocols at https://plos.org/protocols?utm_medium=editorial-email&utm_source=authorletters&utm_campaign=protocols.

As the corresponding author, your ORCID iD is verified in the submission system and will appear in the published article. PLOS supports the use of ORCID, and we encourage all coauthors to register for an ORCID iD and use it as well. Please encourage your coauthors to verify their ORCID iD within the submission system before final acceptance, as unverified ORCID iDs will not appear in the published article. *Only*  the individual author can complete the verification step; PLOS staff *cannot*  verify ORCID iDs on behalf of authors.

We look forward to receiving your revised manuscript.

Kind regards,

Benjamin M. Liu, PhD, D(ABMM), MB(ASCP)

Academic Editor

PLOS One

**Journal Requirements:**

When submitting your revision, we need you to address these additional requirements.

1. Please ensure that your manuscript meets PLOS ONE's style requirements, including those for file naming. The PLOS ONE style templates can be found at

https://journals.plos.org/plosone/s/file?id=wjVg/PLOSOne_formatting_sample_main_body.pdf and

https://journals.plos.org/plosone/s/file?id=ba62/PLOSOne_formatting_sample_title_authors_affiliations.pdf

2. Thank you for stating the following financial disclosure:

“This study was supported by the National Natural Science Foundation of China (Grant No. 82574169), Beijing Nova Programme Interdisciplinary Cooperation Project (Grant No. 20240484724), and High-level Public Health Technical Personnel Construction Project of Beijing Municipal Health Commission (Grant No. Academic leader -03-02).”

Please state what role the funders took in the study.  If the funders had no role, please state: "The funders had no role in study design, data collection and analysis, decision to publish, or preparation of the manuscript."

If this statement is not correct you must amend it as needed.

Please include this amended Role of Funder statement in your cover letter; we will change the online submission form on your behalf.

3. Please note that funding information should not appear in any section or other areas of your manuscript. We will only publish funding information present in the Funding Statement section of the online submission form. Please remove any funding-related text from the manuscript.

4. Your ethics statement should only appear in the Methods section of your manuscript. If your ethics statement is written in any section besides the Methods, please move it to the Methods section and delete it from any other section. Please ensure that your ethics statement is included in your manuscript, as the ethics statement entered into the online submission form will not be published alongside your manuscript.

5. If the reviewer comments include a recommendation to cite specific previously published works, please review and evaluate these publications to determine whether they are relevant and should be cited. There is no requirement to cite these works unless the editor has indicated otherwise.

6. Please review your reference list to ensure that it is complete and correct. If you have cited papers that have been retracted, please include the rationale for doing so in the manuscript text, or remove these references and replace them with relevant current references. Any changes to the reference list should be mentioned in the rebuttal letter that accompanies your revised manuscript. If you need to cite a retracted article, indicate the article’s retracted status in the References list and also include a citation and full reference for the retraction notice.

[Note: HTML markup is below. Please do not edit.]

Reviewers' comments:

Reviewer's Responses to Questions

**Comments to the Author**

1. Does the manuscript provide a valid rationale for the proposed study, with clearly identified and justified research questions?

Reviewer #1: Yes

Reviewer #2: Yes

**********

2. Is the protocol technically sound and planned in a manner that will lead to a meaningful outcome and allow testing the stated hypotheses?

Reviewer #1: Partly

Reviewer #2: Partly

**********

3. Is the methodology feasible and described in sufficient detail to allow the work to be replicable?

Reviewer #1: Yes

Reviewer #2: Yes

**********

4. Have the authors described where all data underlying the findings will be made available when the study is complete?

The PLOS Data policy

Reviewer #1: Yes

Reviewer #2: Yes

**********

5. Is the manuscript presented in an intelligible fashion and written in standard English?

Reviewer #1: Yes

Reviewer #2: Yes

**********

Please use the space provided to explain your answers to the questions above and, if applicable, provide comments about issues authors must address before this protocol can be accepted for publication. You may also include additional comments for the author, including concerns about research or publication ethics.

You may also provide optional suggestions and comments to authors that they might find helpful in planning their study.

Reviewer #1: In the data sets to be analysed, include the comparative analysis of the mortality rates, AMR burden among the serotypes in the study with other serotypes. Include the risk factor analysis for acquisition of such serotypes.

Reviewer #2: Strengths: Strong rationale, clinically important question, PROSPERO registration, integration of systematic review and genomic epidemiology.

Key weaknesses: Limited statistical framework, insufficient ExPEC definition (The protocol states it will include "neonatal ExPEC" isolates but does not clearly specify which definition will be used ) , inadequate consideration of AMR and virulence factors.

Most important gap to address before publication: Expand the protocol to include genomic characterization beyond O-serotyping (particularly antimicrobial resistance genes and major virulence determinants), as these factors are central to neonatal ExPEC epidemiology and vaccine development.

**********

what does this mean?). If published, this will include your full peer review and any attached files.

If you choose “no”, your identity will remain anonymous but your review may still be made public.

**Do you want your identity to be public for this peer review?**  For information about this choice, including consent withdrawal, please see our Privacy Policy

Reviewer #1: **Yes:** Dr Rajat Prakash

Reviewer #2: No

**********

[NOTE: If reviewer comments were submitted as an attachment file, they will be attached to this email and accessible via the submission site. Please log into your account, locate the manuscript record, and check for the action link "View Attachments". If this link does not appear, there are no attachment files.]

To ensure your figures meet our technical requirements, please review our figure guidelines: https://journals.plos.org/plosone/s/figures

You may also use PLOS’s free figure tool, NAAS, to help you prepare publication quality figures: https://journals.plos.org/plosone/s/figures#loc-tools-for-figure-preparation.

NAAS will assess whether your figures meet our technical requirements by comparing each figure against our figure specifications.

# Author comment: Author response to Decision Letter 1

**DOI:** 10.1371/journal.pone.0354124.r002

15 Jun 2026

Response to Reviewers

PONE-D-26-22669

Systematic analysis of global O-serotype prevalence of neonatal extraintestinal pathogenic Escherichia coli: a protocol integrating previous research and bioinformatics databases

Thank you for submitting your manuscript to PLOS ONE. After careful consideration, we feel that it has merit but does not fully meet PLOS ONE’s publication criteria as it currently stands. Therefore, we invite you to submit a revised version of the manuscript that addresses the points raised during the review process.

Reply: Thank you for the opportunity to revise our manuscript. We appreciate the constructive comments provided by the Academic Editor and reviewers. We have carefully considered all suggestions and revised the manuscript accordingly. Detailed responses are provided below, and all changes have been highlighted in the tracked-changes version of the manuscript.

ACADEMIC EDITOR'S COMMENTS:

In the Introduction, the authors are encouraged to introduce the status quo of identification and AST testing for E. coli, the limitations of conventional diagnostic workflows, as bacterial identification and subsequent AST remain time-consuming processes. However, there are some recent advances in novel diagnostic technologies. Lin et al. recently developed a metabolism-driven colorimetric "Read-to-Answer" sensor array for rapid bacterial identification and AST for different bacteria, including E. coli. More references should be cited, with this one (PMID: 40737437) as an example (citing is optional).

Reply: We thank the Academic Editor for this valuable suggestion. We have revised the Introduction to provide additional background on conventional E. coli identification and antimicrobial susceptibility testing (AST) workflows, highlighting their limitations in terms of turnaround time and laboratory requirements. We have also added a brief description of recent advances in rapid diagnostic technologies and cited the recommended reference (PMID: 40737437), together with other relevant literature. These revisions can be found in the Introduction section of the revised manuscript. (See in line 60)

Journal Requirements:

When submitting your revision, we need you to address these additional requirements.

1. Please ensure that your manuscript meets PLOS ONE's style requirements, including those for file naming. The PLOS ONE style templates can be found at

https://journals.plos.org/plosone/s/file?id=wjVg/PLOSOne_formatting_sample_main_body.pdf and

https://journals.plos.org/plosone/s/file?id=ba62/PLOSOne_formatting_sample_title_authors_affiliations.pdf

Reply: Thank you. We have carefully reviewed the manuscript and revised the formatting to comply with all PLOS ONE style requirements, including file naming conventions.

2. Thank you for stating the following financial disclosure:

“This study was supported by the National Natural Science Foundation of China (Grant No. 82574169), Beijing Nova Programme Interdisciplinary Cooperation Project (Grant No. 20240484724), and High-level Public Health Technical Personnel Construction Project of Beijing Municipal Health Commission (Grant No. Academic leader -03-02).”

Please state what role the funders took in the study. If the funders had no role, please state: "The funders had no role in study design, data collection and analysis, decision to publish, or preparation of the manuscript."

If this statement is not correct you must amend it as needed.

Please include this amended Role of Funder statement in your cover letter; we will change the online submission form on your behalf.

Reply: We have added the following statement to the cover letter and request that it be updated in the online submission system: This study was supported by the National Natural Science Foundation of China (Grant No. 82574169), Beijing Nova Programme Interdisciplinary Cooperation Project (Grant No. 20240484724), and High-level Public Health Technical Personnel Construction Project of Beijing Municipal Health Commission (Grant No. Academic leader -03-02). The funders had no role in study design, data collection and analysis, decision to publish, or preparation of the manuscript.

3. Please note that funding information should not appear in any section or other areas of your manuscript. We will only publish funding information present in the Funding Statement section of the online submission form. Please remove any funding-related text from the manuscript.

Reply: Thank you. We have reviewed the manuscript and removed all funding-related information from the main text. Funding information is now provided only in the Funding Statement section of the submission system.

4. Your ethics statement should only appear in the Methods section of your manuscript. If your ethics statement is written in any section besides the Methods, please move it to the Methods section and delete it from any other section. Please ensure that your ethics statement is included in your manuscript, as the ethics statement entered into the online submission form will not be published alongside your manuscript.

Reply: Thank you. We have reviewed the manuscript and ensured that the ethics statement appears only in the Methods section.

5. If the reviewer comments include a recommendation to cite specific previously published works, please review and evaluate these publications to determine whether they are relevant and should be cited. There is no requirement to cite these works unless the editor has indicated otherwise.

Reply: Thank you. We reviewed the recommended publication and considered its relevance to our study. Relevant references have been incorporated where appropriate.

6. Please review your reference list to ensure that it is complete and correct. If you have cited papers that have been retracted, please include the rationale for doing so in the manuscript text, or remove these references and replace them with relevant current references. Any changes to the reference list should be mentioned in the rebuttal letter that accompanies your revised manuscript. If you need to cite a retracted article, indicate the article’s retracted status in the References list and also include a citation and full reference for the retraction notice.

Reply: Thank you. We have carefully reviewed and updated the reference list to ensure completeness and accuracy. No retracted articles were cited in the manuscript.

Reviewers' comments:

6. Review Comments to the Author

Reviewer #1: In the data sets to be analysed, include the comparative analysis of the mortality rates, AMR burden among the serotypes in the study with other serotypes. Include the risk factor analysis for acquisition of such serotypes.

Reply: We thank the reviewer for this valuable suggestion. In response, we have expanded the study outcomes and data extraction framework. Specifically, we have added mortality and other severe clinical outcomes, host and clinical risk factors associated with major O-serotypes, and antimicrobial resistance phenotypes and/or resistance genes as secondary outcomes. Corresponding variables will be extracted where available from published studies and genomic databases. These additions will provide a more comprehensive characterization of neonatal ExPEC epidemiology and strengthen the clinical relevance of the study. (See in line 142-144, 149-152)

Reviewer #2: Strengths: Strong rationale, clinically important question, PROSPERO registration, integration of systematic review and genomic epidemiology.

Key weaknesses: Limited statistical framework, insufficient ExPEC definition (The protocol states it will include "neonatal ExPEC" isolates but does not clearly specify which definition will be used ) , inadequate consideration of AMR and virulence factors.

Most important gap to address before publication: Expand the protocol to include genomic characterization beyond O-serotyping (particularly antimicrobial resistance genes and major virulence determinants), as these factors are central to neonatal ExPEC epidemiology and vaccine development.

Reply: We sincerely thank the reviewer for the constructive comments and positive evaluation of our study. Several revisions have been made accordingly.

First, we have clarified the definition of neonatal ExPEC in the Inclusion and Exclusion Criteria section. For this study, neonatal ExPEC is defined as Escherichia coli isolates recovered from extraintestinal infections in neonates (≤28 days), including bloodstream infections, meningitis, pneumonia, and urinary tract infections. (See in line 105)

Second, we agree that antimicrobial resistance and virulence characteristics are important components of neonatal ExPEC epidemiology. Therefore, we have expanded the secondary outcomes to include the distribution of antimicrobial resistance phenotypes and/or resistance genes, as well as major virulence determinants among predominant O-serotypes, where such information is available from published studies or genomic databases. These additions complement the primary objective of characterizing global O-serotype distribution while maintaining the overall scope and feasibility of the protocol. (See in line 142-144, 149-152)

Attachment Submitted filename: Response to Reviewers.docx

# Editor report: Decision Letter 1

**DOI:** 10.1371/journal.pone.0354124.r003

**Authors:** Benjamin Liu

5 Jul 2026

Systematic analysis of global O-serotype prevalence of neonatal extraintestinal pathogenic Escherichia coli: a protocol integrating previous research and bioinformatics databases

PONE-D-26-22669R1

Dear Dr. Zhang,

We’re pleased to inform you that your manuscript has been judged scientifically suitable for publication and will be formally accepted for publication once it meets all outstanding technical requirements.

Within one week, you’ll receive an e-mail detailing the required amendments. When these have been addressed, you’ll receive a formal acceptance letter and your manuscript will be scheduled for publication.

An invoice will be generated when your article is formally accepted. Please note, if your institution has a publishing partnership with PLOS and your article meets the relevant criteria, all or part of your publication costs will be covered. Please make sure your user information is up-to-date by logging into Editorial Manager at Editorial Manager® and clicking the ‘Update My Information' link at the top of the page. For questions related to billing, please contact billing support.

If your institution or institutions have a press office, please notify them about your upcoming paper to help maximize its impact. If they’ll be preparing press materials, please inform our press team as soon as possible -- no later than 48 hours after receiving the formal acceptance. Your manuscript will remain under strict press embargo until 2 pm Eastern Time on the date of publication. For more information, please contact onepress@plos.org.

Kind regards,

Benjamin M. Liu, PhD, D(ABMM), MB(ASCP)

Academic Editor

PLOS One

Additional Editor Comments (optional):

Reviewers' comments:

# Editor report: Acceptance letter

**DOI:** 10.1371/journal.pone.0354124.r004

**Authors:** Benjamin Liu

PONE-D-26-22669R1

PLOS One

Dear Dr. Zhang,

I'm pleased to inform you that your manuscript has been deemed suitable for publication in PLOS One. Congratulations! Your manuscript is now being handed over to our production team.

At this stage, our production department will prepare your paper for publication. This includes ensuring the following:

* All references, tables, and figures are properly cited

* All relevant supporting information is included in the manuscript submission,

* There are no issues that prevent the paper from being properly typeset

You will receive further instructions from the production team, including instructions on how to review your proof when it is ready. Please keep in mind that we are working through a large volume of accepted articles, so please give us a few days to review your paper and let you know the next and final steps.

Lastly, if your institution or institutions have a press office, please let them know about your upcoming paper now to help maximize its impact. If they'll be preparing press materials, please inform our press team within the next 48 hours. Your manuscript will remain under strict press embargo until 2 pm Eastern Time on the date of publication. For more information, please contact onepress@plos.org.

You will receive an invoice from PLOS for your publication fee after your manuscript has reached the completed accept phase. If you receive an email requesting payment before acceptance or for any other service, this may be a phishing scheme. Learn how to identify phishing emails and protect your accounts at https://explore.plos.org/phishing.

If we can help with anything else, please email us at customercare@plos.org.

Thank you for submitting your work to PLOS One and supporting open access.

Kind regards,

PLOS ONE Editorial Office Staff

on behalf of

Dr. Benjamin M. Liu

Academic Editor

PLOS One
